# Supplementary material for: Design and Validation of a Multi-Point Injection Technology for MR-Guided Convection Enhanced Delivery in the Brain
Source: Front Med Technol. 2021 Oct 14;3:725844. doi: 10.3389/fmedt.2021.725844 (PMC8757778; doi:10.3389/fmedt.2021.725844)
Supplement: Supplementary file 1 [file Data_Sheet_1.docx]

**Supplementary Figures**

**Design and validation of a multi-point injection technology for MR-guided convection enhanced delivery in the brain**

Kayla Prezelski,^1,2,3^ Megan Keiser,^4^ Joel Stein,^5^ Timothy H. Lucas,^2,6^ Beverly Davidson,^4,7^ Pedro Gonzalez-Alegre,^4,8^ Flavia Vitale^1,2,3,8,9^*

1. Department of Bioengineering, University of Pennsylvania, Philadelphia, Pennsylvania 19104, USA
2. Center for Neuroengineering and Therapeutics, University of Pennsylvania, Philadelphia, Pennsylvania 19104, USA
3. Center for Neurotrauma, Neurodegeneration, and Restoration, Corporal Michael J. Crescenz Veterans Affairs Medical Center, Philadelphia, PA 19104, USA
4. Raymond G. Perelman Center for Cellular and Molecular Therapeutics, The Children’s Hospital of Philadelphia, Philadelphia, PA 19104, USA
5. Department of Radiology, Perelman School of Medicine, University of Pennsylvania, Philadelphia, Pennsylvania 19104
6. Department of Neurosurgery, Perelman School of Medicine, University of Pennsylvania, Philadelphia, Pennsylvania 19104
7. Department of Pathology and Laboratory Medicine, University of Pennsylvania, Philadelphia, PA 19104, USA
8. Department of Neurology, Perelman School of Medicine, University of Pennsylvania, Philadelphia, Pennsylvania 19104
9. Department of Physical Medicine and Rehabilitation, University of Pennsylvania, Philadelphia, 19104

*Corresponding author


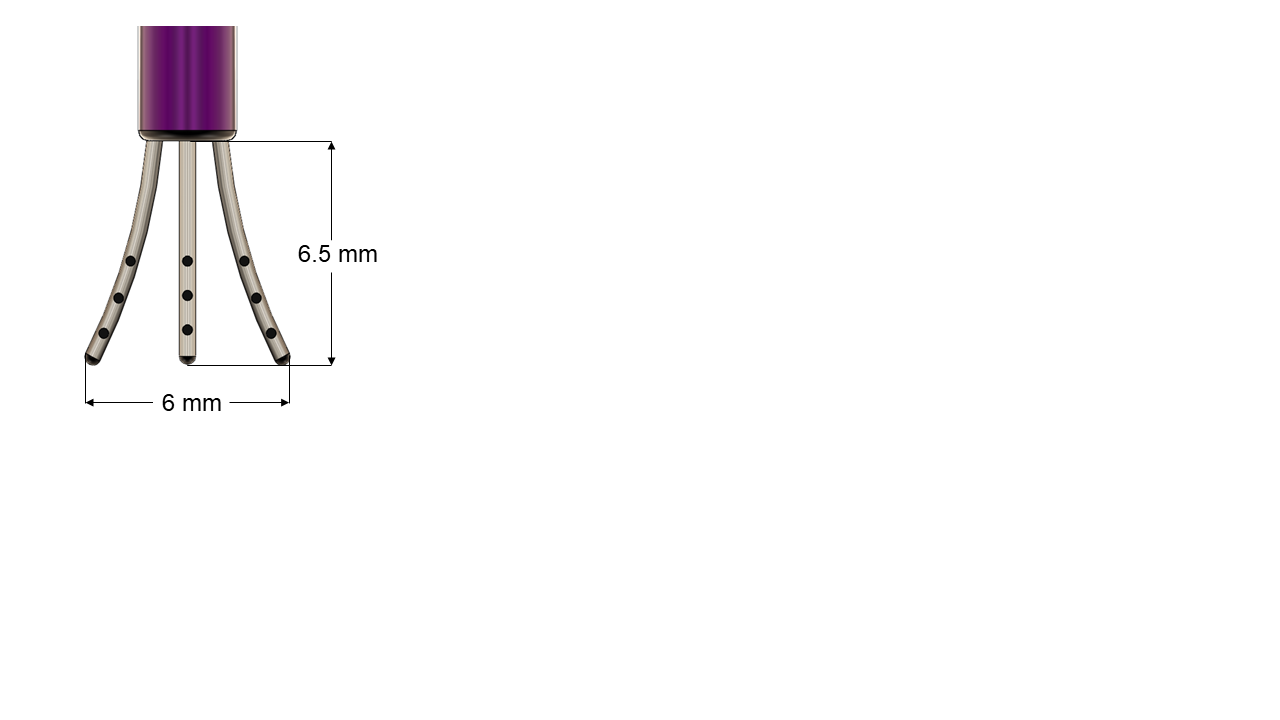


**Supplementary Figure 1.** Overview of the dimensions of the MINT catheter showing the ejected microcannula position of the device designed for infusions in non-human primates.


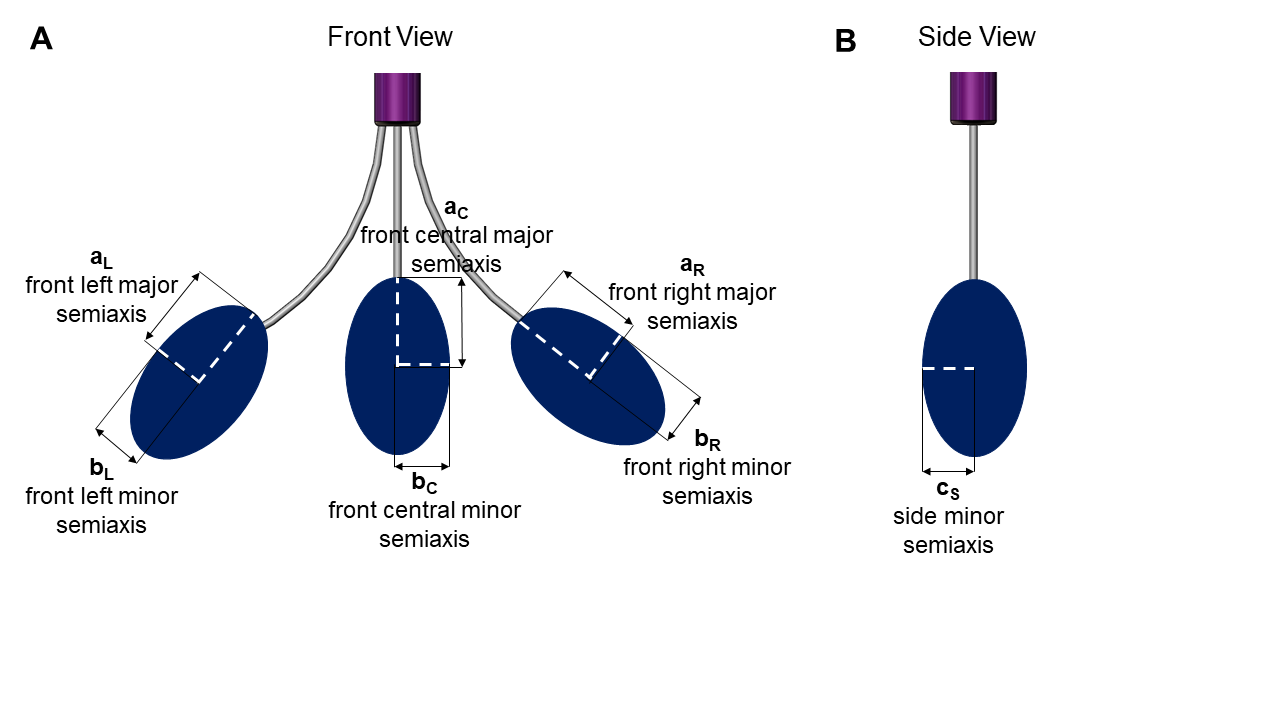


**Supplementary Figure 2.** Volume distribution was calculated as the sum of the volume of the three ellipsoids according to the formula V=4/3𝜋abc. (**A**) The major and minor semiaxes of the front view and (**B**) the minor semiaxis of the side (mirror) view were used in the calculations as shown.


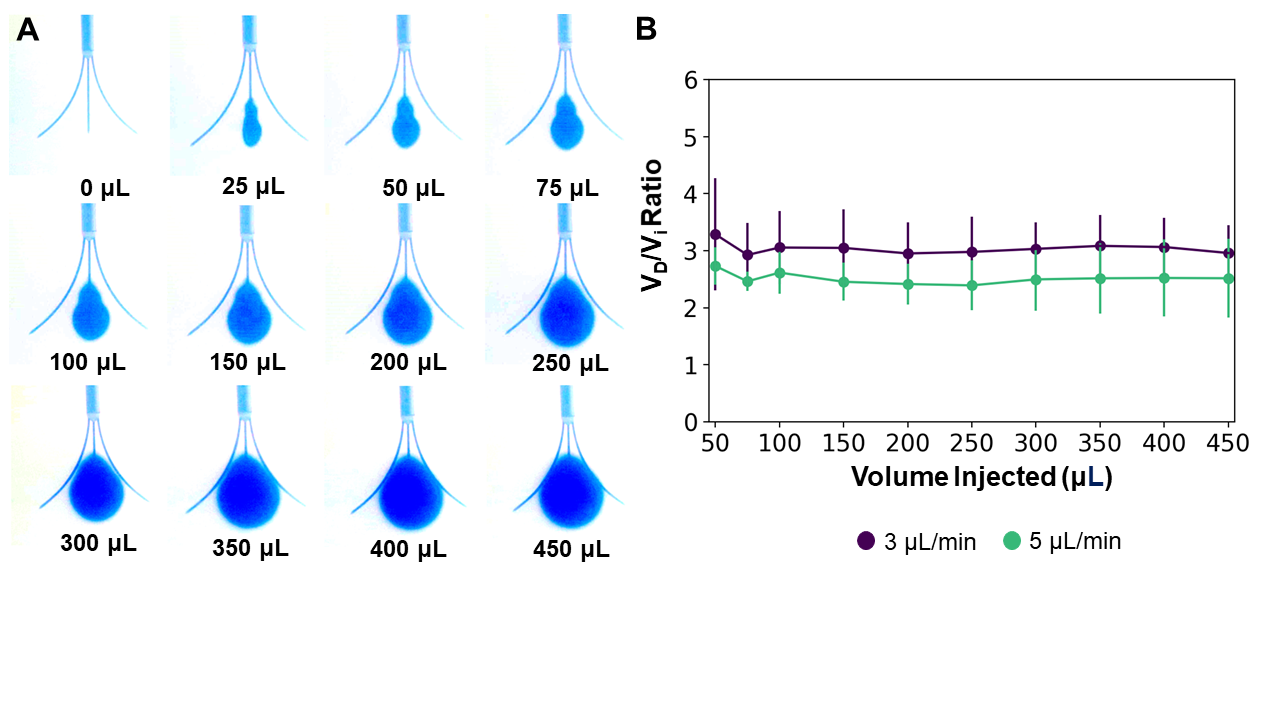


**Supplementary Figure 3.** (**A**) Snapshots of the volume distribution V_D_ during single point CED injections from only the MINT central microcannula. (**B**) Average V_D_/V_i_ at from the MINT central microcannula at 3 and 5 µL/min total flow rate. Error bars represent S.D. for n=4 trials.
